# First Evidence of a Volatile Sex Pheromone in Lady Beetles

**DOI:** 10.1371/journal.pone.0115011

**Published:** 2014-12-16

**Authors:** Bérénice Fassotte, Christophe Fischer, Delphine Durieux, Georges Lognay, Eric Haubruge, Frédéric Francis, François J. Verheggen

**Affiliations:** 1 Functional and Evolutionary Entomology, Gembloux Agro-Bio Tech, University of Liege, Gembloux, Belgium; 2 Unit of Analysis Quality and Risk, Laboratory of Analytical Chemistry, Gembloux Agro-Bio Tech, University of Liege, Gembloux, Belgium; University of Paris 13, France

## Abstract

To date, volatile sex pheromones have not been identified in the Coccinellidae family; yet, various studies have suggested that such semiochemicals exist. Here, we collected volatile chemicals released by virgin females of the multicolored Asian lady beetle, *Harmonia axyridis* (Pallas), which were either allowed or not allowed to feed on aphids. Virgin females in the presence of aphids, exhibited “calling behavior”, which is commonly associated with the emission of a sex pheromone in several Coleoptera species. These calling females were found to release a blend of volatile compounds that is involved in the remote attraction (i.e., from a distance) of males. Gas Chromatography-Mass Spectrometry (GC-MS) analyses revealed that (–)-β-caryophyllene was the major constituent of the volatile blend (ranging from 80 to 86%), with four other chemical components also being present; β-elemene, methyl-eugenol, α-humulene, and α-bulnesene. In a second set of experiments, the emission of the five constituents identified from the blend was quantified daily over a 9-day period after exposure to aphids. We found that the quantity of all five chemicals significantly increased across the experimental period. Finally, we evaluated the activity of a synthetic blend of these chemicals by performing bioassays which demonstrated the same attractive effect in males only. The results confirm that female *H. axyridis* produce a volatile sex pheromone. These findings have potential in the development of more specific and efficient biological pest-control management methods aimed at manipulating the behavior of this invasive lady beetle.

## Introduction

Many insect species rely on sex pheromones to communicate for courtship and mating [1]. In general, sex pheromones are produced by females to attract males; however, a small proportion of these sex attractants are emitted by males [2], as documented for several Lepidoptera species, including *Bicyclus anynana* Butler [3] and *Pieris napi* L. [4], and for the Cerambycid beetle, *Hedypathes betulinus* Klug [5].

To date, data about lady beetle sex pheromones, or any other semiochemicals that play a role in Coccinellid mating, remain limited [6], [7]. However, behavioral observations of lady beetles [8]–[12] and the existence of sexual dimorphism in olfactory receptors of some species [13] have led several authors to hypothesize that these types of molecules are involved in sexual communication. For instance, the elytra and abdomen extracts of female *Coleomegilla maculata* (De Geer) induce a significant response from the antennae of conspecific males [14]. In addition, male *Leptothea galbula* (Mulsant) are strongly attracted to quiescent female pupae, and may remain with the pupae until emergence [15]. It has been suggested that this behavior is induced by females producing a sex pheromone at both adult and pupal stages. The sex pheromone has been predicted to act as a locomotion inhibitor once contact is established between male and pupa [15]. Furthermore, males of *Propylea dissecta* (Mulsant) were found to copulate with recently dead females (up to 7 days), whereas these females were neglected one month after death [16]. In this work, Omkar and Ahmad suggested that mating semiochemicals remain after death, and progressively decay with time. *H. axyridis* males were also found to copulate with freshly dead females, indicating that gender specific chemical cues were present on the surface of female bodies [8]. Although visual and tactile cues might also be involved in mate recognition, these chemical cues appeared to be the key stimulus for a copulatory attempt. Sex recognition might be due to a difference in the cuticular chemical substances present on the body surface; however, direct contact would be required between individuals to detect it as no remote attraction was observed [8].

Therefore, all previous studies conducted on sex recognition mediated by chemical cues in the Coccinellidae family have focused on short range non-volatile cues [17]. To our knowledge, no study has demonstrated the existence of a volatile sex pheromone that is involved in the remote attraction of lady beetles. Yet, a field study highlighted that aphid infestations alone could not explain the distribution pattern of *H. axyridis* [18]. In this preceding study, groups were primarily composed of a mixture of both sexes, and males were never observed alone, indicating that a chemical signal might regulate the distribution pattern of lady beetles. The hypothesis of a mutual attraction has been excluded because of sex-related differences in behavior. For instance, females rest or feed, whereas males are highly agitated, even when they enter an aphid colony. This behavior might be interpreted as mate-searching behavior [18], and could be associated to the production of a volatile sex pheromone by females.

In this work, we set out to record whether virgin female lady beetles exhibit the typical “calling behavior” that has been previously associated with the emission of a sex pheromone in several other Coleoptera species. Subsequently, volatile collection was performed to identify and quantify the chemical components of the emitted blend, and the behavioral response of conspecifics was tested. The findings of this study are expected to expand knowledge about sex pheromone production in the Coccinellidae family, and would contribute towards improving biological control methods involving *H. axyridis.*

## Materials and Methods

### Biological material

#### 1. Wild lady beetles

Pupae of *H. axyridis* were collected from corn fields in the vicinity of Gembloux (Belgium) during the summer of 2012 (end of July to mid-August) and at the end of May 2014. No specific permits were required for the described field studies, as *H. axyridis* is not an endangered or protected species. The pupae were separately housed in aerated plastic Petri dishes (diameter: 4 cm; height: 1 cm), until they emerged. Three days after emergence, lady beetles acquire their final pigmentation and the gender may be determined using morphological characteristics, as described by McCornack *et al*. [19]. Males and females of the same age were then identified and placed by gender into 36×15×8 cm aerated plastic boxes (±20 individuals per container). Sugar lumps were placed in each box, in addition to a water-impregnated sponge and bee-collected multiflower pollen. Both Petri dishes and plastic boxes were placed in controlled environment chambers, with a 16 h-light photoperiod, 24±1°C temperature, and 45±15% relative humidity (RH); males and females were placed in separate chambers to avoid chemical communication through the aeration of the boxes. As *H. axyridis* requires 7–10 days to reach sexual maturity [20], the experiments were conducted on the adults one month after emergence. To avoid the morphotype having any potential effect on the emission profile of the sex pheromone, only *succinea* individuals (i.e., red elytra with or without black spots) were used in the experiments.

#### 2. Plant and aphid rearing

In a climate-controlled room (16-h light photoperiod; 50±10% RH; 24±2°C), pea aphids, *Acyrthosiphon pisum* (Harris), were bred on host-plants (*Vicia faba* L.) grown in 30×20×6 cm plastic containers filled with a mixture of vermiculite and perlite (1/1). The plants were replaced weekly to guarantee the proper development of aphids. To do so, old stems infested with *A. pisum* were cut and transferred into containers with undamaged plants.

*Vicia faba* plants intended for use in the headspace sampling procedure and biological assays were grown in 7×7×10 cm plastic pots containing the previously described mixture of vermiculite and perlite (1/1). Five seeds were sown in each pot, and were supplied with water daily. Plants were allowed to grow for two weeks before the onset of the experiments. The climatic conditions of the culture chamber were maintained at 24±1°C and 45±15% RH, with a 16 h-light photoperiod.

### Headspace sampling procedure

A dynamic sampling technique was used to collect volatile organic compounds released by *H. axyridis*. The compounds were collected from the headspace of glass chambers containing either 15 virgin females or 15 males over 15 consecutive days. Lady beetles were fed sugar lumps for the first 5 days in the presence of a pot of *V. faba* plants (see section 2.1.2 for description). On the sixth day, the sugar lumps were removed from the chambers, and the plants were manually infested with 4 g of *A. pisum*. Every two days, the same quantity of pea aphids was reintroduced, so that lady beetles were fed *ad libitum*. Aphids were provided to *H. axyridis* adults because (1) their consumption triggers sexual maturation and (2) females oviposit in habitats where high densities of aphid prey are located [18]. Headspace sampling was also performed in a control glass chamber (same content as the other glass chambers, but lady beetles were absent) to verify the origin of volatile compounds.

A purified airstream was blown into each 4 L glass chamber (inner diameter: 12 cm; height: 36 cm). The airstream was purified by being passed through activated charcoal filter cartridges with a digital pump at a flow rate of 700 ml/min. The airflow was checked with an airflow meter every day during the sampling period. The volatile compounds that were released were trapped on adsorbent cartridges containing 30 mg of a copolymer of ethylvinylbenzene and divinylbenzene (80–100 mesh). A security cartridge containing 60 mg of the same adsorbent was also added to detect any breakthrough. All of the tubing connections of the collection system were made entirely of PTFE. The collection of volatiles was conducted 24 h/day. The system was only turned off for less than 5 min/day to replace the cartridges and to refill the chambers with aphids (every 2 days). The experiment was conducted in a laboratory that was free from any semiochemical use, at 22±2°C temperature and 50±10% RH. Three replicates were performed for the control and for both sexes.

After collection, the trapped volatile compounds were eluted from each adsorbent cartridge with 200 µl *n*-hexane (HPLC grade, purity>95%). A quantity of 1 µl internal standard at 9 µg/ml was added, and each elution sample was capped into chromatography vials.

### Volatile compound identification and quantification

To compare the chemical profiles, male and female headspace samples were analyzed by GC-MS. To do so, 1 µl of each extract was injected onto a non-polar capillary column (5% diphenyl; 30 m×0.25 mm I.D.; film thickness 0.25 µm). The operating conditions were: splitless, injector at 240°C; carrier gas: helium at a constant flow rate of 1.0 ml/min. The oven temperature program was initiated at 40°C, held for 2 min, then raised to 4°C/min to 95°C, and then raised by increments of 6°C/min to 155°C, and finally raised by increments of 25°C/min to 280°C, and was held at this temperature for 5 min. The mass spectra were recorded in the electron impact mode at 70 eV (source at 200°C, transfer line at 250°C, scanned mass range: 39 to 300 m/z). The detected peaks were identified from their retention data, and by comparing the obtained mass spectra with those from spectral libraries. Retention indices were determined using the retention time of *n*-alkane standards (from C_9_ to C_40_, 10 µg/ml in *n*-hexane), and compared against literature values. The identification of volatile compounds of interest was then confirmed by injecting commercial standards of high purity (>98%). The calibration method for the quantification of the volatile compounds was completed for a concentration range of (–)-β-caryophyllene in *n*-hexane, according to the validated method described by Heuskin *et al*. [21]. (–)-β-Caryophyllene was extracted by flash chromatography from catnip (*Nepeta cataria* L.) essential oil with 98% purity, following the fractionation process described by Heuskin *et al*. [22]. *n*-Butylbenzene (purity>99.9%) was used as internal standard at 10 µg/ml at each concentration level. To generate the calibration curve, five standard solutions (from 5 µg/ml to 600 µg/ml of (–)-β-caryophyllene) and blanks were injected as data points, and analyzed in three replicates. The injections were repeated twice more for the central point of the curve (i.e., the 60 µg/ml solution). The calibration curve was obtained by plotting the ratio of analyzed peak area/internal standard peak area versus the analyte concentration. The method of least squares fit analysis was used to calculate the calibration curve. The linearity was considered satisfactory when the correlation coefficient was higher than 0.996.

To determine the emission profile of lady beetles, the amounts of each daily-collected volatile compound were quantified by gas chromatography (GC). The device was equipped with a flame ionization detector (FID at 260°C) and a non-polar capillary column (5% phenyl; 30 m×0.25 mm I.D.; film thickness 0.25 µm). Splitless injections were performed with 1 µl of each extract. The injector temperature was 250°C; the carrier gas was helium (1 ml/min). The temperature program was 40°C for 2 min, then an increase at increments of 10°C/min to 300°C, with this final temperature being held for 5 min. Compounds were quantified with *n*-butylbenzene as the internal standard (9 µg/ml). Quantification was performed on each sample collected from females and for the three replicates of the volatile collection procedure.

### Bioassays

#### 1. H. axyridis females placed in glass chambers containing plants and aphids

To test the hypothesis that virgin females emit a sex pheromone, behavioral assays were conducted. To do so, *H. axyridis* individuals were confronted with the volatile organic compounds released by females in a two-arm olfactometer. The device was similar to the four-arm olfactometer previously described by Vet *et al*. [23]; however, two opposite arms were closed during the experiments. Charcoal-filtered air was blown through PTFE tubing in the other two olfactometer arms, with digital pumps operating at a flow rate of 150 ml/min. The central walking arena was ventilated by removing the air at a rate of 300 ml/min. During bioassays, a 4 L glass chamber (inner diameter: 12 cm; height: 36 cm) was connected to each arm as an odor source. One arm was connected to a glass chamber containing a pot of *V. faba* plants manually infested with 4 g of pea aphids and 20 *H. axyridis* females. The other arm was connected to a control glass chamber that only contained the plants infested with the same quantity of aphids. Glass chambers were randomly connected to one of the two olfactometer arms during each experiment.

The device was divided into one central squared zone (length: 10 cm), which was considered as the neutral zone, and two other zones that were connected to different odor sources. The choice of 30 males and 30 females was determined by (a) the first entered zone, (b) the zone where the lady beetle stayed for the longest period of time, and (c) the last entered zone (i.e., the zone where the lady beetle was observed after three minutes). The neutral zone was not included as an option and any lady beetle remaining in this zone for the entire duration of the test was excluded. The experiment duration was fixed at 3 min, which was sufficient for individuals to explore the entire experimental set-up. Males and females were introduced randomly into the olfactometer and each lady beetle was only used once. The walking arena and the glass ceiling were both cleaned with *n*-hexane after each experiment. After testing 30 lady beetles, the olfactometer was rotated by 180 degrees to avoid any bias toward one of the arms. All of the experiments were carried out in a laboratory at 24±1°C temperature, 45±5% RH, and under uniform lighting.

#### 2. Extracts from H. axyridis females

Natural extracts from females fed with aphids were produced to ensure that the potential attractive effect was due to the volatile compounds they release, rather than chemical cues resulting from interactions between plants, aphids and lady beetles. To do so, females *H. axyridis* previously fed with aphids were crushed into test tubes by adapting the method detailed in Fischer and Lognay [24]. Extractions were performed without solvent and the volatile compounds emitted by a single female were trapped on 30 mg adsorbent cartridges by active sampling. Incoming air was blown at a flow rate of 700 ml/min and was purified with activated charcoal filter cartridges. The collection of volatiles was conducted over a three–hour period. Adsorbent cartridges were then eluted as explained in section 2.3. and samples were analyzed by GC-MS to obtain the chemical profile of the extracts (see operating conditions in section 2.4.).

Finally, the extract activity was tested in the two arm olfactometer, by using the same protocol as that described in section 2.5.1. The lady beetles were confronted with a natural extract (crushed female fed with aphids) and a control extract (crushed female fed with pollen and sugar) as odor sources. For each replicate, fresh extracts were made and these odor sources were randomly assigned to one of the olfactometer arms. Each tested lady beetle was only used once.

#### 3. Synthetic blend

A synthetic blend was prepared by combining the identified compounds emitted by females in *n*-hexane. The relative amount of each component was calculated according to day 14 of the emission profile (S1 Tables) so that 200 µl of the solution was equivalent to the concentration of volatiles emitted by one individual. GC-MS analyses were then performed to confirm the chemical profile of the synthetic blend (see operating conditions in section 2.4.).

*H. axyridis* responses to the blend were evaluated by performing behavioral experiments in dual-choice olfactometry. Odor sources consisted of filter papers (diameter: 5 cm) loaded with 200 µl of synthetic blend or *n*-hexane (control). Each tested lady beetle was only used once. After each replicate, the filter papers were replaced and the odor sources were randomly assigned to one of the olfactometer arms. The protocol was identical to that previously described in section 2.5.1.

### Statistical analyses

The observed frequencies related to the choice of lady beetles in the olfactometer were analyzed by using a two-sided binomial test based on the null hypothesis that the probability of choice for the odor source or the control is equal to 50%.

## Results

### Initial observations

During the headspace sampling experiment, virgin females exhibited distinctive behavior. Three days after the lady beetles consumed aphids, they rhythmically extended and contracted their abdomens, while raising their elytra above their bodies. As a result, concealed portions of some tergites were exposed. The head and thorax were lowered to the support. These movements (on both horizontal and vertical surfaces) were generally repeated several times. Occasionally, some females probed the surface of the glass chamber with the tip of their abdomen, and ejected a small drop of fluid. This typical behavior contains important similarities to what has been termed “calling behavior” in other Coleoptera species.

On the 13th day of the volatile collection procedure (i.e., 7 days after consuming the aphids), *H. axyridis* females started to lay eggs. The eggs were incubated at the end of the experiment, but did not hatch because they were unfertilized.

### Volatile compound identification and quantification

**Figure 1:**
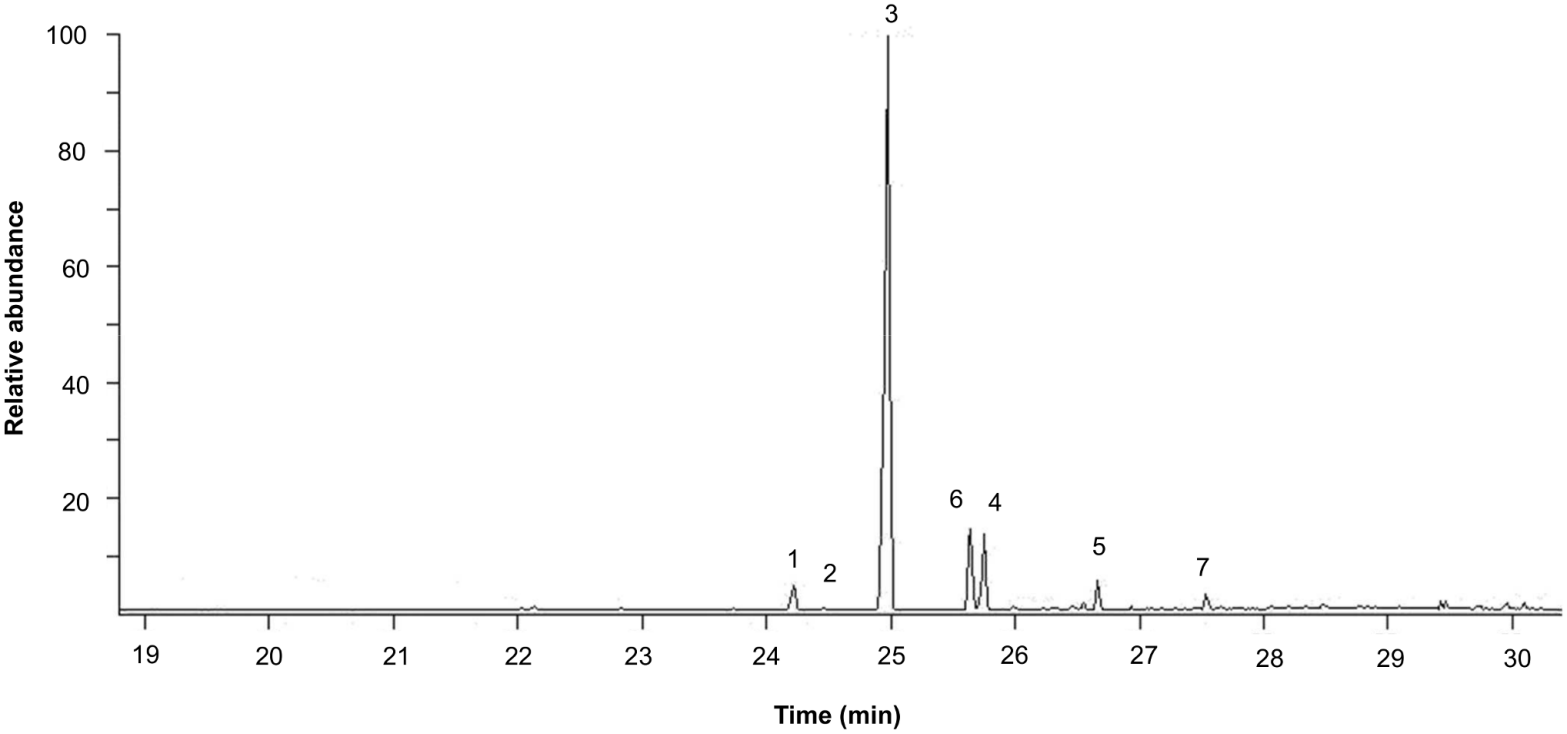
Gas chromatogram of the sex pheromone components emitted by *H. axyridis* females. The chromatogram was obtained on the 12th day of sampling. The peaks that were associated with the pheromonal blend are numbered according to the components identified by GC-MS in S1 Table. These components were identified as β-elemene (1), methyl-eugenol (2), (–)-β-caryophyllene (3), α-humulene (4), and α-bulnesene (5). Peak 6 is (E)-β-farnesene, which was emitted by aphids, and peak 7 is an unknown compound of plant origin.

Five volatile compounds were identified from the headspace of virgin females that had been fed for 3 days with aphids, whereas males did not produce any of these molecules. The volatile cues emanating from the females included (–)-β-caryophyllene, β-elemene, methyl-eugenol, α-humulene, and α-bulnesene (see Fig. 1 for a chromatogram that was obtained on the 12th day of sampling). As shown in the chromatogram, the main constituent was (–)-β-caryophyllene, with lower proportions of the other four compounds being detected. The Kovats indices calculated for the five components are presented in S1 Table.

The linearity of the calibration curve was validated with a correlation coefficient (r^2^) higher than 0.996 (reference coefficient) (S1 Figure). Within the indicated concentration range (i.e., from 5 µg/ml to 600 µg/ml of (–)-β-caryophyllene in *n*-hexane), a good correlation was obtained between the peak area and the analyte concentration.

**Figure 2:**
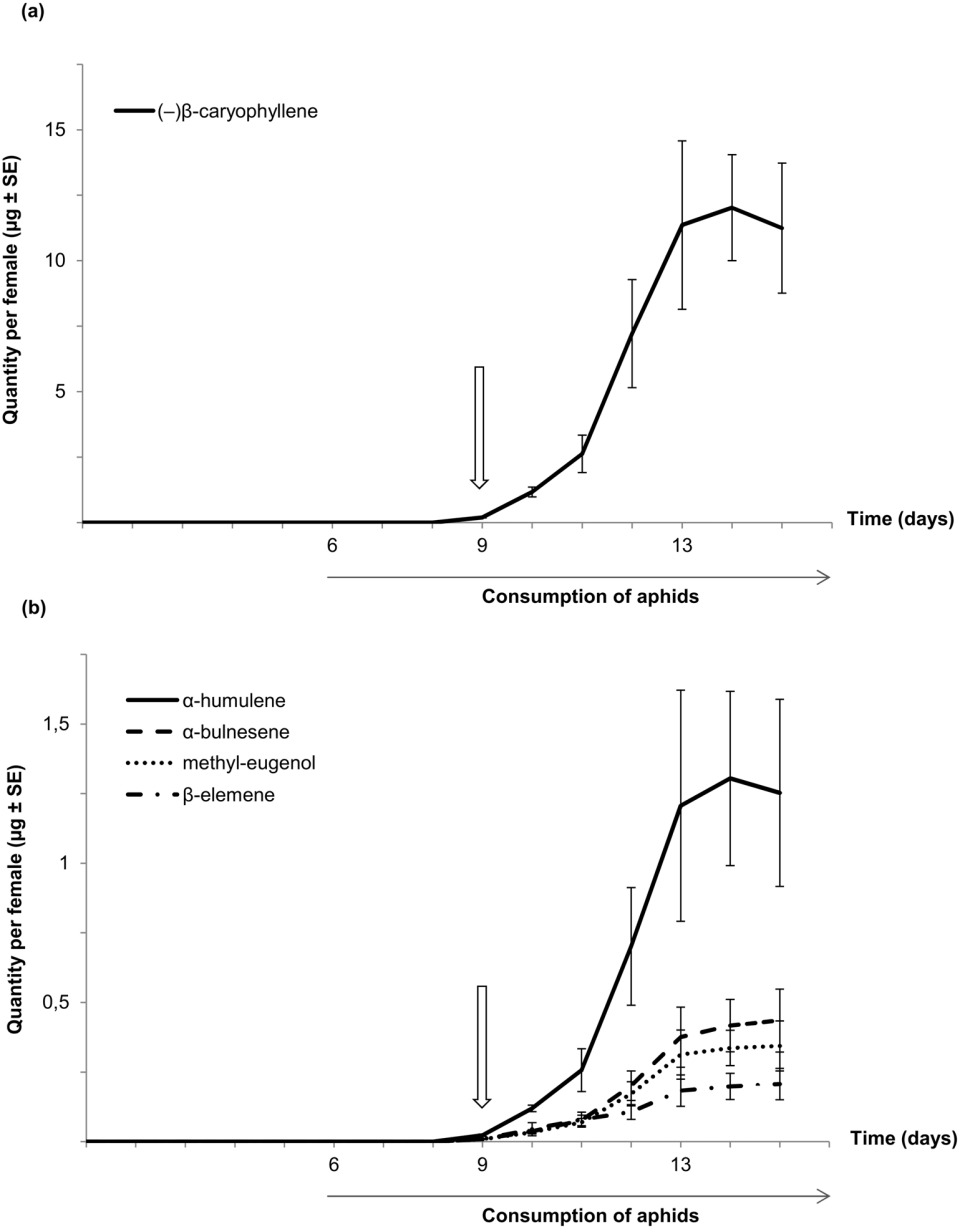
Emission profile of the sex pheromone. The quantities (µg) per virgin female are expressed as (–)-β-caryophyllene equivalent on a daily basis (mean ± SE of three replicates). Panel (a) corresponds to (–)-β-caryophyllene which is depicted separately from the other four compounds because of the large difference in terms of quantities (see ordinate axes). Panel (b) represents the emission profile of β-elemene, methyl-eugenol, α-humulene, and α-bulnesene at a different scale. Downward pointing arrows indicate the onset of calling behavior which coincides with pheromone emission on the ninth day.

The emission profile of the blend was established over a period of 15 consecutive days by gas chromatography. As shown in Fig. 2, *H. axyridis* females started to produce volatile cues 3 days after exposure to aphids. Then, the quantities of volatile compounds gradually increased over time, reaching a threshold on the 14th day for (–)-β-caryophyllene and α-humulene. The quantity of the other three compounds (β-elemene, methyl-eugenol, and α-bulnesene) continued to gradually rise during this period. However, all the five compounds produced a similar emission profile, but were present in different proportions in the blend. The major constituent was (–)-β-caryophyllene, representing between 80 and 86% of the total blend. The quantities emitted by each female over time are summarized in S1 Table.

### Bioassays

**Figure 3:**
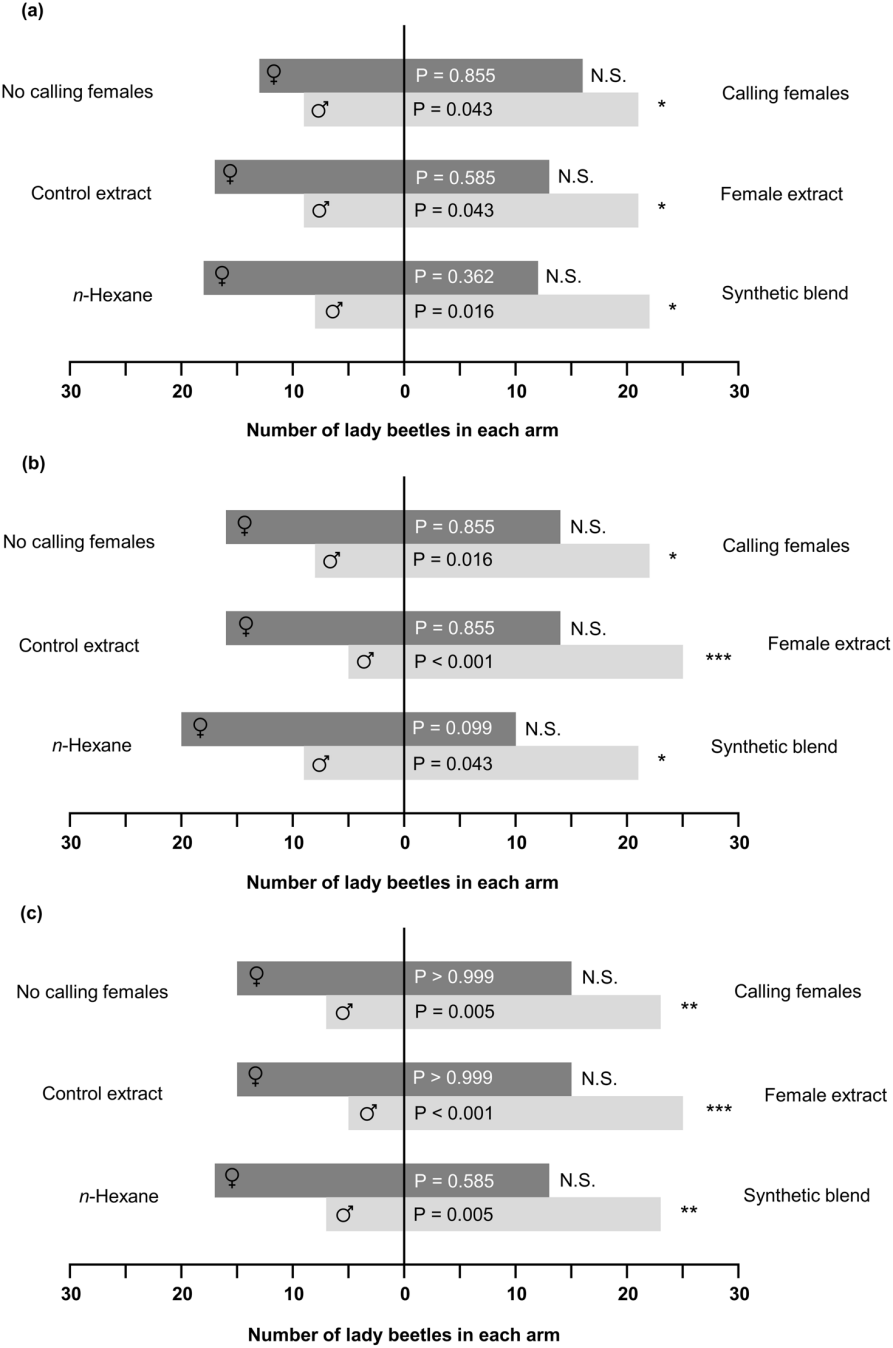
Behavioral responses of *H. axyridis* toward the tested odor sources. The choice between the two arms was observed for individual male and female ladybeetles (n = 30 for both genders in each of the three sets of experiments), in relation with (a) the first entered zone, (b) the zone where the lady beetle stayed for the longest period of time, and (c) the last entered zone. Statistical differences are indicated by asterisks at P<0.05 (*), P<0.01 (**), and P<0.001 (***) respectively, whereas N.S. indicates no significant difference (two-sided binomial test).

We conducted three sets of experiments to evaluate the potential attractive effect of the semiochemicals released by virgin *H. axyridis* females. In all three bioassays, we found that the volatile compounds released by (1) *H. axyridis* females placed in glass chambers containing plants and aphids; (2) extracts from *H. axyridis* females; (3) a synthetic blend; elicited significant behavioral responses in males only (Fig. 3).

Furthermore, the chemical profile of the extracts made from females and the chemical profile of the synthetic blend were similar, their composition consisting exclusively of the volatile compounds emanating from virgin females that had been fed with aphids.

## Discussion

In the current study, we confirmed that, in the presence of aphid prey, virgin females of the multicolored Asian lady beetle emit a sex pheromone, and demonstrated that males are able to perceive and respond to this pheromone. In addition, we characterized the contents of the pheromone, with this information being of potential use for developing biological pest-control methods.

GC-MS analyses revealed that virgin females fed with *A. pisum* emitted specific volatile cues, whereas males did not produce any of these compounds. Five components were identified from the headspace samples of females with the main component being (–)-β-caryophyllene, while lower quantities of four other molecules were also detected: β-elemene, methyl-eugenol, α-humulene, and α-bulnesene. The identification of all the compounds was based on spectral libraries and retention indices, and was subsequently confirmed by injections of standards, except for α-bulnesene, which is not commercially available.

The behavioral experiments demonstrated that the volatile cues produced by virgin females served as a sex pheromone, only attracting males. The results of the bioassays associated with the synthetic blend are similar to those obtained with biological material (*H. axyridis* females or volatile extracts from *H. axyridis* females), even if α-bulnesene, which is not commercially available, was not included in the blend. However, the presence of this chemical in appropriate proportions could strengthen the attractive effect and the results significance. The semiochemical (–)-β-caryophyllene is already known to induce behavioral responses in various insect species. Indeed, it acts as an attractant for the green lacewing (*Chrysoperla carnea* Stephens) [25], for the western corn rootworm (*Diabrotica virgifera virgifera* Le Conte) [26], and for the carrot fly (*Psila rosae* Fabricius) [27]. In addition, (–)-β-caryophyllene plays a role in the host location process in the black bean aphid, *Aphis fabae* Scop. [28], and stimulates oviposition in the alfalfa seed wasp, *Bruchophagus roddi* Gussakovski [29]. A previous study related to lady beetles, documented the involvement of (–)-β-caryophyllene in the aggregation process of overwintering individuals, and showed that only *H. axyridis* females emit this molecule [30]. The production of this compound by females was confirmed in the current study by the collection of volatiles and subsequent GC-MS analyses. However, (–)-β-caryophyllene was found to act as a component of the sex pheromone in this species that was involved in the remote attraction of males. This result is consistent with those obtained by Verheggen *et al*. [31], who reported the same attractive effect on males, with some females also exhibiting slightly significant responses. In contrast, wind-tunnel assays conducted by Leroy *et al*. demonstrated that this semiochemical attracted female *H. axyridis*, whereas males did not respond to this cue [32]. The mismatch between the present study and previous research might be explained by the different concentrations of (–)-β-caryophyllene that were tested; specifically, the pure compound from chemical synthesis [31], chemical formulation in paraffin oil [32], or, in this case, as a component of a natural sex pheromone. Indeed, insect behavioral responses are known to vary according to the concentration of the volatile being applied [14]. Furthermore, an electroantennographic bioassay highlighted the existence of neuronal receptors allowing the perception of (–)-β-caryophyllene in this species, and showed that male antennae were more sensitive to this semiochemical compared to female antennae [31].

When analyzing the emission profile, we highlighted that virgin females start to emit volatile cues 3 days after being fed *A. pisum*. The chemical analyses revealed that all of the compounds exhibited a similar emission profile, whereby their quantities gradually increased across the sampling period. This observation strongly supports that (–)-β-caryophyllene, β-elemene, methyl-eugenol, α-humulene, and α-bulnesene are part of the pheromonal blend.

This work provides the first evidence confirming that sexually receptive females exhibit a characteristic behavior. This behavior contains important similarities to what has been previously termed “calling behavior”, which has been described for several Coleoptera species in the Cerambicydae family [33]–[35] and the Dermestidae family [36], in addition to cockroaches [37]. The female calling behavior of many insect species is usually associated with the release of a volatile sex pheromone [38]–[40].

The synchronized production of chemicals and behavioral responses was also obtained for the multicolored Asian lady beetle in the current study. The calling behavior was observed 3 days after the lady beetles were fed aphids, which directly coincides with the onset of pheromone emission. To complete our observations, it would be interesting to analyze the calling periodicity, and to collect the secreted fluid immediately after emisssion. The fluid and its components could be analyzed, which would enable us to elucidate the fluid function (pheromone diffuser or substrate marking for a suitable site to lay eggs, for example) through ethological tests. In addition, the relationship between calling behavior and the virgin status requires examination to determine whether mated or gravid females still display this behavior. A negative result would indicate that calling plays a role in mate-finding by this species.

To date, the organ responsible for sex pheromone production in the multicolored Asian lady beetle has yet to be elucidated. It is very likely that specific abdominal glands are involved in the production process, as observed for other Coleoptera species [41], [42] and for the German cockroach, *Blattela germanica* L. [43]. Moreover, previous research on the eleven-spotted lady beetle, *Semiadalia undecimnotata* Schneider, demonstrated that glands with and without secretory ducts are distrubuted over the head, thorax, and abdomen. Glands without ducts were thought to release a volatile pheromone [44].

The results presented in this study constitute the first evidence of a volatile sex pheromone in lady beetles. It would be interesting to investigate the existence of such semiochemicals in other Coccinellid species to analyze the specificity of the pheromone. Some of the compounds detected in the pheromonal blend of *H. axyridis*, might also be found in other lady beetle species, but in different proportions or combined with other volatile substances. This phenomenon has been previously documented for many aphid species, in which (E)-β-farnesene is the major constituent of the alarm pheromone [45].

The current study provides baseline information that could promote the development of more specific and efficient biological pest-control management methods. A first approach would consist in manipulating the movements of *H. axyridis* in fields. For instance, the potential attractive effect of this pheromone could be useful in the development of push-pull strategies involving the multicolored Asian lady beetle as a biological control agent against aphids. However, because this invasive lady beetle has negative impacts on biodiversity [46], the sex pheromone might also be used for mass trapping leading to less ecological damage when populations tend to increase. In conclusion, this study provides important biological information regarding the sexual communication in *H. axyridis*, with potential application to pest-control management systems.

## Supporting Information

S1 Figure **Calibration curve of (–)-β-caryophyllene obtained by the least squares fit analysis method.** Peak area ratio is presented as the analyte peak area on an internal standard peak area.
(TIF)

S1 Table **Identification and relative amount of volatile compounds emitted by virgin** ***H. axyridis*** **females.** The daily samples were obtained from the headspace of glass chambers containing 15 virgin females (n = 3 replicates).
(DOCX)
