# Supplementary material for: First Evidence of a Volatile Sex Pheromone in Lady Beetles
Source: PLoS One. 2014 Dec 16;9(12):e115011. doi: 10.1371/journal.pone.0115011 (PMC4267778; doi:10.1371/journal.pone.0115011)
Supplement: S1 Table — Identification and relative amount of volatile compounds emitted by virgin H. axyridis females. The daily samples were obtained from the headspace of glass chambers containing 15 virgin females (n = 3 replicates). (DOCX) [file pone.0115011.s002.docx]

Table S1. Identification and relative amount of volatile compounds emitted by virgin *H. axyridis* females. The daily samples were obtained from the headspace of glass chambers containing 15 virgin females (n = 3 replicates).

| **Peak number ^(a)^** | **Name** | **KI ^(c)^** | **RT ^(d)^** | **Quantities (µg) of volatile compounds emitted per female and per day (expressed as (–)-β-caryophyllene equivalent) (mean ± SE of 3 replicates)** | | | | | | | |
| --- | --- | --- | --- | --- | --- | --- | --- | --- | --- | --- | --- |
|  |  |  |  | **Day 1 - 8** | **Day 9** | **Day 10** | **Day 11** | **Day 12** | **Day 13** | **Day 14** | **Day 15** |
| 1 | β-elemene ^(b)^ | 1389 | 24.21 | ND | 0.008 ± 0.001 | 0.045 ± 0.023 | 0.079 ± 0.027 | 0.107 ± 0.026 | 0.183 ± 0.057 | 0.198 ± 0.047 | 0.207 ± 0.056 |
| 2 | Methyl-eugenol ^(b)^ | 1398 | 24.43 | ND | 0.008 ± 0.001 | 0.034 ± 0.006 | 0.069 ± 0.014 | 0.172 ± 0.043 | 0.312 ± 0.088 | 0.336 ± 0.063 | 0.344 ± 0.090 |
| 3 | (–)-β-caryophyllene ^(b)^ | 1425 | 24.98 | ND | 0.189 ± 0.011 | 1.167 ± 0.187 | 2.622 ± 0.712 | 7.214 ± 2.065 | 11.359 ± 3.219 | 12.024 ± 2.025 | 11.242 ± 2.485 |
| 4 | α-humulene ^(b)^ | 1463 | 25.75 | ND | 0.023 ± 0.004 | 0.119 ± 0.011 | 0.257 ± 0.077 | 0.701 ± 0.211 | 1.207 ± 0.415 | 1.305 ± 0.313 | 1.252 ± 0.336 |
| 5 | α-bulnesene | 1515 | 26.67 | ND | 0.009 ± 0.001 | 0.037 ± 0.006 | 0.076 ± 0.019 | 0.201 ± 0.053 | 0.375 ± 0.108 | 0.417 ± 0.094 | 0.435 ± 0.113 |

(a) The peak number correspond to the peaks identified on the chromatogram (Figure 1)

(b) Identification confirmed by the injection of commercial standards

(c) Kovats indices calculated for the compounds identified by GC-MS

(d) Retention time of the compounds identified by GC-MS

ND, Not detected
